# Identification of a drought stress response module in tomato plants commonly induced by fungal endophytes that confer increased drought tolerance

**DOI:** 10.1007/s11103-024-01532-y

**Published:** 2024-12-17

**Authors:** Adrián González Ortega-Villaizán, Eoghan King, Manish K. Patel, Estefanía Rodríguez-Dobreva, Marcia González-Teuber, Patricio Ramos, Jesús Vicente-Carbajosa, Begoña Benito, Stephan Pollmann

**Affiliations:** 1 Centro de Biotecnología y Genómica de Plantas, Universidad Politécnica de Madrid (UPM)-Instituto Nacional de Investigación y Tecnología Agraria y Alimentación (INIA/CSIC), Campus de Montegancedo, Pozuelo de Alarcón, Madrid, Spain; 2 Departamento de Genética Molecular y Microbiología, Facultad de Ciencias Biológicas, Pontifica Universidad Católica de Chile, Santiago, Chile; 3 Plant Microorganism Interaction Laboratory, Instituto de Ciencias Biológicas, Universidad de Talca, Talca, Chile; 4 Departamento de Biotecnología-Biología Vegetal, Escuela Técnica Superior de Ingeniería Agronómica, Alimentaria y de Biosistemas, Universidad Politécnica de Madrid (UPM), Madrid, Spain

**Keywords:** Drought stress, Endosymbiosis, Plant–microbe interactions, *Solanum lycopersicum*, Transcriptional regulation

## Abstract

Global climate change exacerbates abiotic stresses, as drought, heat, and salt stresses are anticipated to increase significantly in the coming years. Plants coexist with a diverse range of microorganisms. Multiple inter-organismic relationships are known to confer benefits to plants, including growth promotion and enhanced tolerance to abiotic stresses. In this study, we investigated the mutualistic interactions between three fungal endophytes originally isolated from distinct arid environments and an agronomically relevant crop, *Solanum lycopersicum*. We demonstrated a significant increase in shoot biomass under drought conditions in co-cultivation with *Penicillium chrysogenum* isolated from Antarctica, *Penicillium minioluteum* isolated from the Atacama Desert, Chile, and *Serendipita indica* isolated from the Thar Desert, India. To elucidate plant gene modules commonly induced by the different endophytes that could explain the observed drought tolerance effect in tomato, a comprehensive transcriptomics analysis was conducted. This analysis led to the identification of a shared gene module in the fungus-infected tomato plants. Within this module, gene network analysis enabled us to identify genes related to abscisic acid (ABA) signaling, ABA transport, auxin signaling, ion homeostasis, proline biosynthesis, and jasmonic acid signaling, providing insights into the molecular basis of drought tolerance commonly mediated by fungal endophytes. Our findings highlight a conserved response in the mutualistic interactions between endophytic fungi isolated from unrelated environments and tomato roots, resulting in improved shoot biomass production under drought stress.

**Supplementary Information:**

The online version contains supplementary material available at 10.1007/s11103-024-01532-y.

## Key message

Despite their genetic and evolutionary distance, two fungal endophytes from the Thar Desert in India, *Serendipita indica*, and Antarctica, *Penicillium chrysogenum*, trigger a common transcriptional response in tomato plants to confer increased drought tolerance.

**Supplementary Information:**

The online version contains supplementary material available at 10.1007/s11103-024-01532-y.

## Introduction

Plants are continuously subjected to changes in their environment, including biotic stresses, such as pathogen infections or herbivores, and abiotic stresses, such as drought, heat, cold, flooding, salinity, metal toxicity, or nutrient deficiency, which impact crop productivity. The severity of these abiotic stresses is exacerbated by the current climate change scenario and is projected to increase significantly in the coming years (Kumar 2016). Water deficit is a major abiotic stress that severely affects crop yields (Barnabás et al. 2008). The climate change report of the Intergovernmental Panel on Climate Change (IPCC) indicates that heatwaves and droughts are anticipated to increase in frequency in the coming years (IPCC 2023). Plants coexist with a diverse array of microorganisms, both detrimental and beneficial. Since the initial definition of symbiotic interactions (de Bary 1879) and the subsequent refinement of the interpretation (Hertig et al. 1937), plant–microbe interactions have been recognized to confer numerous benefits for plant performance in terms of growth promotion and abiotic stress tolerance (Rodriguez et al. 2004). Plant endophytes are conventionally defined as microorganisms residing within plant tissues, both aboveground and belowground, capable of existing for the majority or the entirety of their life cycle inside the plant without causing harm (Stone et al. 2000). However, the concept of plant endophyte has been redefined more recently by Hardoim et al. (2015) as microbes that colonize and inhabit plant tissues regardless of the outcome of the interaction. In this study, *Penicillium chrysogenum*, *Penicillium minioluteum*, and *Serendipita indica*, three root colonizing fungal endophytes isolated from extremely arid environments, have been investigated. *Serendipita indica* (formerly known as *Piriformospora indica*) is an axenically root colonizing endophyte of the order Sebacinales, isolated from the Thar Desert in India, with a broad host range (Verma et al. 1998; Weiss et al. 2016; Mensah et al. 2020). The strain of *P. chrysogenum* utilized in this investigation was isolated in Antarctica from the roots of the vascular plant *Colobanthus quitensis* (Oses-Pedraza et al. 2020). This strain has been characterized as a root colonizing fungal endophyte capable of colonizing the roots of *Lactuca sativa* and *S. lycopersicum* (Molina-Montenegro et al. 2020). The *P. minioluteum* strain employed in this study originates from the Atacama Desert in Chile, where it was isolated from *Chenopodium quinoa* roots (González-Teuber et al. 2018). Numerous studies have previously investigated the effects of these fungal endophytes on plant performance. For instance, *S. indica* promotes plant performance and biomass production (Varma et al. 1999; Peškan-Berghöfer et al. 2004; Vadassery et al. 2009; De Rocchis et al. 2022; Pérez-Alonso et al. 2022), and enhances tolerance to biotic and abiotic stresses in its host plant (Jogawat et al. 2016; Sefloo et al. 2019; Tsai et al. 2020; Shukla et al. 2022). *Penicillium chrysogenum* has also been reported to promote plant growth and abiotic stress tolerance (Molina-Montenegro et al. 2020; Morsy et al. 2020; Morales-Quintana et al. 2022), while *P. minioluteum* enhances salt stress tolerance in soybean (Khan et al. 2011) and drought stress and salt stress tolerance in quinoa (González-Teuber et al. 2018, 2022). The ability of endophytes to promote growth or enhance stress tolerance of their host plants is hypothesized to be a consequence of either direct or indirect mechanisms (Santoyo et al. 2016). They can facilitate nutrient acquisition, produce secondary metabolites, or modulate the levels or signaling pathways of phytohormones (Waqar et al. 2024). Phytohormones are key drivers in plant stress responses. They are well-established small signaling molecules that rapidly change in their abundance in response to environmental changes and act at sub-micromolar concentrations (Davies 2010). The classical five phytohormone classes include auxins, gibberellins, abscisic acid (ABA), ethylene, and cytokinins (Gaspar et al. 1996). However, jasmonic acid (JA), brassinosteroids, and salicylic acid (SA) have subsequently been added to this classification (Bari and Jones 2009). ABA, JA, and SA are recognized as the primary stress phytohormones in plants, due to their roles in plant stress responses.

According to recent studies, *S. indica* enhances drought tolerance. It has been demonstrated that *S. indica* enhances water stress tolerance in rice by regulating stomatal behavior and reactive oxygen species (ROS) scavenging systems (Tsai et al. 2020). Stomatal aperture and associated physiological processes are largely controlled by ABA (Davies 2010). Furthermore, *S. indica* promotes proline accumulation in walnut roots in response to drought stress (Liu et al. 2021). It has also been observed that the *P5CS* gene related to proline biosynthesis is induced in tomato inoculated with *S. indica* under drought stress conditions (Azizi et al. 2021).

In this study, we investigated the drought tolerance phenotype of tomato plants inoculated with three genetically unrelated fungal endophytes from diverse extreme environments to address the question of the existence of shared response modules in plants commonly activated by beneficial root-colonizing fungal symbionts. To this end, a comprehensive transcriptomics analysis of tomato roots under drought conditions, inoculated with either *P. chrysogenum*, *P. minioluteum*, or *S. indica*, followed by a weighted gene co-expression network analysis (WGCNA) was performed. The experiments enabled us to identify a core drought response module in tomato that is induced by at least two fungi isolated from the Antarctic region and the Thar Desert in India. The module contains genes related to ABA, auxin, and ion homeostasis that are differentially expressed in the roots upon fungal infection.

## Materials and methods

### Plant material and growth conditions

In this study, *S. lycopersicum* cv. Moneymaker was employed. The tomato seeds underwent surface sterilization with 70% v/v ethanol for 2 min, 50% v/v sodium hypochlorite from a commercial bleach solution (4% w/v) for 15 min and were subsequently rinsed with sterile H_2_O five times. The sterilized seeds were then germinated on sterilized filter paper with autoclaved milliQ water in darkness at 22 °C for 4 days. Following germination, seedlings were individually transferred to pots containing a mixture of peat:vermiculite (3:1), where they were cultivated in the greenhouse at 24 °C with a photoperiod of 16 h. The endophytic fungi utilized in this investigation were as follows: *P. chrysogenum* (GenBank accession number KJ881371) isolated from *Colobanthus quitensis* in Antarctica (Oses-Pedraza et al. 2020), *P. minioluteum* (recently reclassified as *Talaromyces minioluteus*) (GenBank accession number HM771268.1) isolated from quinoa plants originating from the Atacama Desert, Chile (González-Teuber et al. 2018). These strains were provided by the laboratory of Dr. Patricio Ramos. Moreover, *S. indica* strain DSM 11827 was used. It was obtained from the German Collection of Microorganisms and Cell Cultures (DSMZ) in Braunschweig, Germany. The fungi were cultivated on Potato Dextrose Agar (PDA) medium at a constant temperature of 28 °C and sub-cultured weekly.

### Field capacity drought assay

To investigate differences in biomass production in inoculated plants, a field capacity (FC) drought experiment was conducted. 1100 g of dry mixtures of peat:vermiculite (3:1) were placed in 25 cm diameter pots. To determine 100% FC and 40% FC, the pots were saturated with water and drained for 2 days. The pots were then weighed, establishing 100% FC at 2379 g and 40% FC at 1683 g. After transferring 1 tomato seedling per pot with 100% FC, the seedlings were inoculated with 100 ml of spore-containing water twice with an interval of 2 weeks between the inoculations. The inoculation concentration for the different fungi was 5 × 10^5^ spores ml^−1^. After transferring the seedlings into soil, the 100% FC condition pots were readjusted to 100% FC every 2 days; whereas the 40% FC condition pots were not watered until they reached 40% FC. Following the second inoculation, the plants were cultivated for 6 weeks, with FC readjustment every 2 days.

After 6 weeks, stomatal conductance was measured in fully expanded mature leaves using a steady-state leaf porometer (SC-1, Decagon Devices, LabFerrer, Spain). Additionally, physiological parameters, including the height of the aerial part and its fresh weight (FW) were measured. To determine the dry weight (DW), the aerial plant parts were placed in a Venticell® type desiccator for 3 days at 90 °C and weighed again.

### Trypan blue staining and root fungal isolation experiments

To assess the root colonization capacity of the examined fungi, tomato seedlings were cultivated in square Petri dishes containing Plant Nutrition Medium (PNM) (Johnson et al. 2013), and the roots were inoculated with 20 µl of spore solution at a concentration of 2 × 10^5^ spores ml^−1^. After 7 days of growth under strictly controlled environmental conditions (16 h light, 8 h darkness, constant temperature of 22 °C, 100 to 105 µmol photons m^−2^ s^−1^ photosynthetically active radiation), 10 to 12 small root samples from control and infected plants were selected. After thorough washing of the root samples with deionized water, they were sectioned into 1 cm long pieces and incubated overnight in 10 N KOH. The explants were subsequently rinsed 5 times with sterile H_2_O, before incubation for 5 min in 0.1 N HCl. Subsequently, the samples were incubated in a 0.05% Trypan blue solution (w/v), before being partially decolorized with lactophenol for 10 min. Prior to mounting the specimens on glass slides and examining them by microscopy, they were washed once with 100% ethanol, 3 times with sterile H_2_O, and stored in 60% glycerol (v/v).

To re-isolate the endophytes from roots, the tissue was surface sterilized with 70% ethanol (v/v) for 1 min, followed by an incubation in sodium hypochlorite 2% (v/v) for 4 min and rinsing with sterile H_2_O. After washing the roots, they were immersed in 70% ethanol (v/v) for 30 s, washed 3 times with sterile H_2_O and dried on a piece of autoclaved Whatman paper. As a surface sterilization control, all roots were wiped over a PDA plate. Finally, the roots were sectioned into 0.5–1 cm pieces and placed on a PDA plate, followed by an incubation for 6 days in darkness at 28 °C.

### RNA isolation and gene expression analysis by RT-qPCR

For the RT-qPCR experiments, a total of 24 RNA extractions were conducted. For each water regime (100% FC and 40% FC), mock-treated, *P. chrysogenum*-, *P. minioluteum*-, and *S. indica*-infected roots were sampled in triplicate. For each sample, 300 mg of root tissue from tomato plants 6 weeks post-infection were harvested for total RNA extraction as previously described (Oñate-Sánchez and Vicente-Carbajosa 2008). First-strand synthesis was conducted utilizing M-MLV reverse transcriptase and oligo(dT)_15_ primer, in accordance with the manufacturer's instructions (Promega, Madison, WI, USA). 2 ng of cDNA were utilized as template in each RT-qPCR. cDNA amplification was performed employing the FastStart SYBR Green Master solution (Roche Diagnostics, Basel, Switzerland) and a Lightcycler 480 Real-Time PCR system (Roche Diagnostics, Basel, Switzerland), in accordance with the supplier's instructions. Relative transcript quantification was calculated utilizing the comparative 2^−ΔΔCT^ method (Livak and Schmittgen 2001). As the tomato reference gene, *UBIQUITIN3* (*UBI3*) was employed (Hoffman et al. 1991). Refer to Supplementary Table S1 for primer sequences.

### RNA-Seq analysis

In this study, a genome-wide expression analysis was conducted utilizing mRNA sequencing (RNA-Seq). Initially, total RNA was extracted from 6-week-old control and fungus-inoculated roots as previously described and quantified using a Nanodrop ND-1000® UV/Vis spectrophotometer (ThermoFisher, Waltham, MA, USA). RNA quality was subsequently assessed on a Bioanalyzer 2100 (Agilent, Santa Clara, CA, USA) by the Novogene Genomics Service (Cambridge, UK). The experiments were conducted in triplicate. Library construction and sequencing (150-nt paired-end reads) on Illumina NovaSeq™ 6000 platforms were also performed by the Novogene Genomics Service, which additionally provided basic data analysis employing their RNA-Seq pipeline. The reference genome for tomato was ensemblplants_solanum_lycopersicum_sl3_0_gca_000188115_3. Sequence reads underwent quality assessment using FastQC (v. 0.11.3) and were mapped against the reference genome with HISAT2.

The analysis of the RNA-Seq data was conducted using the R-Shiny application DIANE (Cassan et al. 2021). To identify differentially expressed genes (DEGs), a false discovery rate (FDR) < 0.05 and a log_2_ (fold change) ≥|1| were applied. To illustrate the commonalities and differences between the DEGs of the different datasets, Venn diagrams were generated using the Venn (http://bioinformatics.psb.ugent.be/webtools/Venn/) online tool.

### Weighted gene co-expression network analysis

Weighted gene co-expression network analysis (WGCNA) was conducted utilizing the WGCNA-Shiny application (https://github.com/ShawnWx2019/WGCNA-shinyApp?tab=readme-ov-file#wgcna-shinyapp) in R. Gene counts were normalized employing the Deseq2::vst method. Features with consistently low normalized counts (norm. count < 20 in more than 90% of the samples) were excluded. The secondary filtration method was based on mean expression, with the number of retained genes set at 5000. A signed hybrid network (power β = 14) was generated. A minimum module size of 30 and a module cut-tree height of 0.25 were selected. Eigengene-based connectivity (kME) and the corresponding p-value were calculated for the 5000 genes clustered in 7 modules. The gene ontology (GO) classifications for *S. lycopersicum* are currently limited compared to the model plant *Arabidopsis thaliana*. Consequently, ortholog genes in *A. thaliana* were identified and utilized using g:Orth from the g:Profiler online tool (Kolberg et al. 2023). For tomato genes with multiple associated Arabidopsis orthologs, the Arabidopsis protein exhibiting the highest sequence similarity to the tomato protein was selected using the blastp tool from the EnsemblPlants database (https://plants.ensembl.org/index.html). Subsequent GO enrichment analysis was performed using the AgriGO v2.0 online tool (Tian et al. 2017). To reduce redundant GO terms, the online tool ReviGO (Supek et al. 2011) was employed. The principal component analysis and dot plots were generated using the Shiny-PCA-Maker tool (https://github.com/LJI-Bioinformatics/Shiny-PCA-Maker) in R. The heatmap for representing gene expression was constructed with TBtools (Chen et al. 2020). Functional relationships between differentially expressed genes (DEGs) were analyzed using stringApp v1.7 (Doncheva et al. 2019) in Cytoscape v3.10.1 (Shannon et al. 2003).

### Statistical analysis

The statistical analysis of the data was conducted using GraphPad Prism 8.0 software (GraphPad Software, Inc., La Jolla, CA, United States). Student's t-test was utilized to compare two means. Results were deemed statistically significant when the p-value was less than 0.05.

## Results

### The tested fungi enhance biomass production in tomato under drought conditions

The effects of fungal endophytes on plants under abiotic stress are extensively studied. *Serendipita indica* confers tolerance to drought in maize (Zhang et al. 2018), tomato (Azizi et al. 2021) and barley (Ghabooli et al. 2013). *Penicillium minioluteum* is reported to confer salt stress tolerance in soybeans (Khan et al. 2011), and *P. chrysogenum* confers drought and salt stress tolerance in tomato (Morsy et al. 2020).

In this study, the drought stress alleviating effects of these three different fungal endophytes on *S. lycopersicum* plants were investigated. Initially, the capacity of the fungal endophytes to infect the tomato seedlings was assessed to ensure a comparable test system. Through re-isolation experiments and trypan blue staining, it was confirmed that *S. indica*, *P. chrysogenum*, and *P. minioluteum* were capable of infecting the roots of *S. lycopersicum* seedlings (Supplementary Fig. S1).

**Fig. 1:**
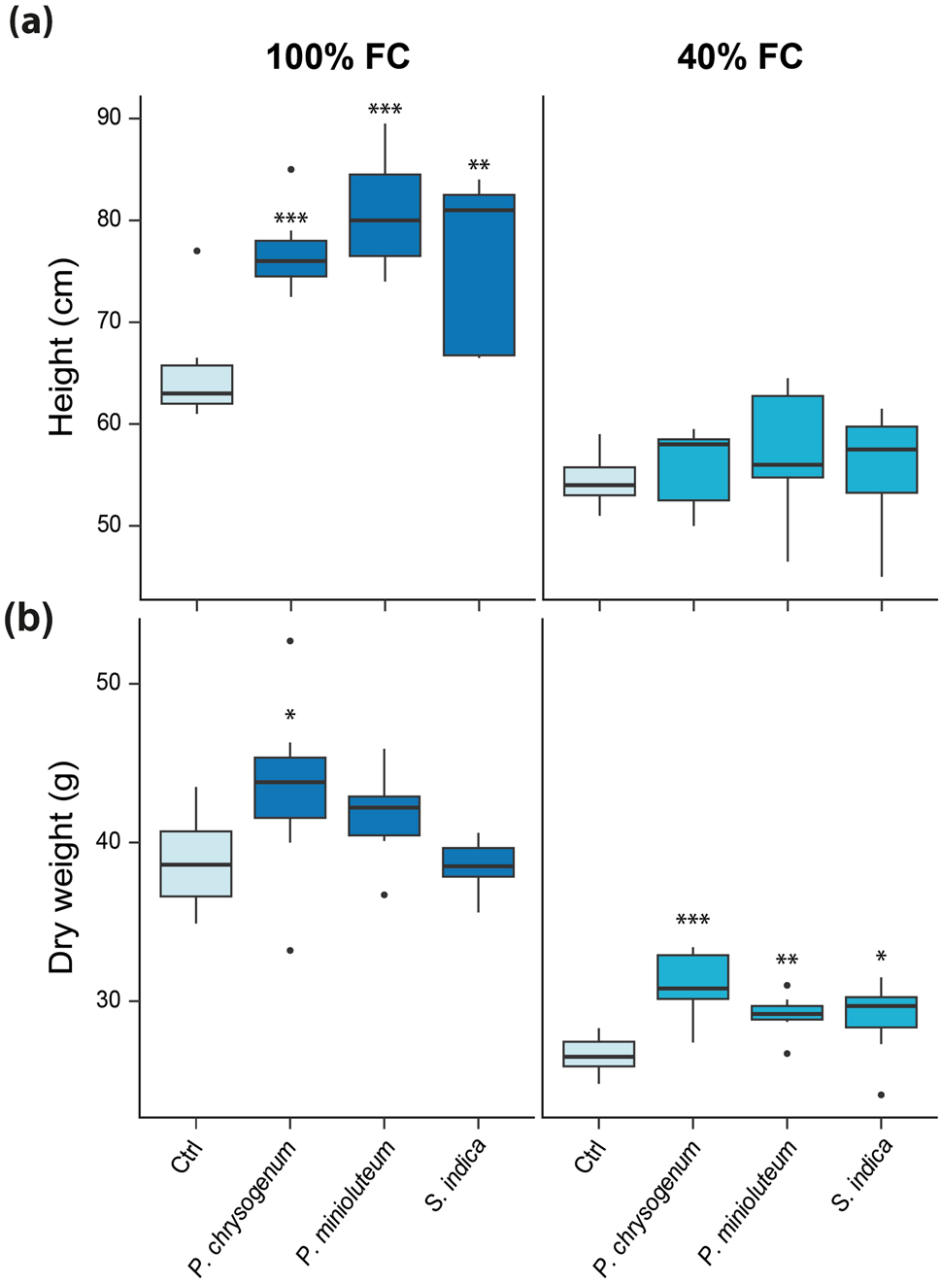
Physiological measurements of *S. lycopersicum* 6 weeks after mock (Ctrl) and fungus treatment. **a** Boxplots of plant height*.* **b** Boxplot of aboveground organs dry weight. The boxes show means of n = 7 independent measurements. Asterisks mark the genes with a significantly altered expression. Student’s *t*-test: *p ≤ 0.05, ** p ≤ 0.01, ***p ≤ 0.001

Six weeks after the second infection and drought treatment, several physiological measurements were conducted. As illustrated in Fig. 1a, significant differences in plant height were observed in watered plants inoculated with the different fungal endophytes. Notably, the values obtained under drought conditions did not exhibit significant differences. Regarding biomass, after measuring the dry weight of the aerial parts, significant differences were observed between watered plants inoculated with *P. chrysogenum* and plants under drought conditions inoculated with *P. chrysogenum*, *P. minioluteum*, and *S. indica* (Fig. 1b).

In addition to height and dry weight, stomatal conductance and fresh weight were also analyzed (Supplementary Fig. S2). Stomatal conductance was measured to verify the effects of the applied drought conditions. The applied stress conditions were confirmed, as the stomatal conductance under drought stress (40% FC) was significantly lower than under the control conditions (100% FC). Given the significant dry weight enhancement observed in the aerial parts of inoculated plants under drought conditions compared to control plants, we subsequently conducted an RNA-Seq analysis to elucidate the molecular mechanism(s) underlying the observed increased biomass in endophyte-inoculated plants. Considering the root endophytic lifestyle of the three fungi, root tissue from mock-treated and fungus-infected plants was harvested and analyzed 6 weeks post-infection.

### Transcriptional response to drought stress and fungal infection

**Fig. 2:**
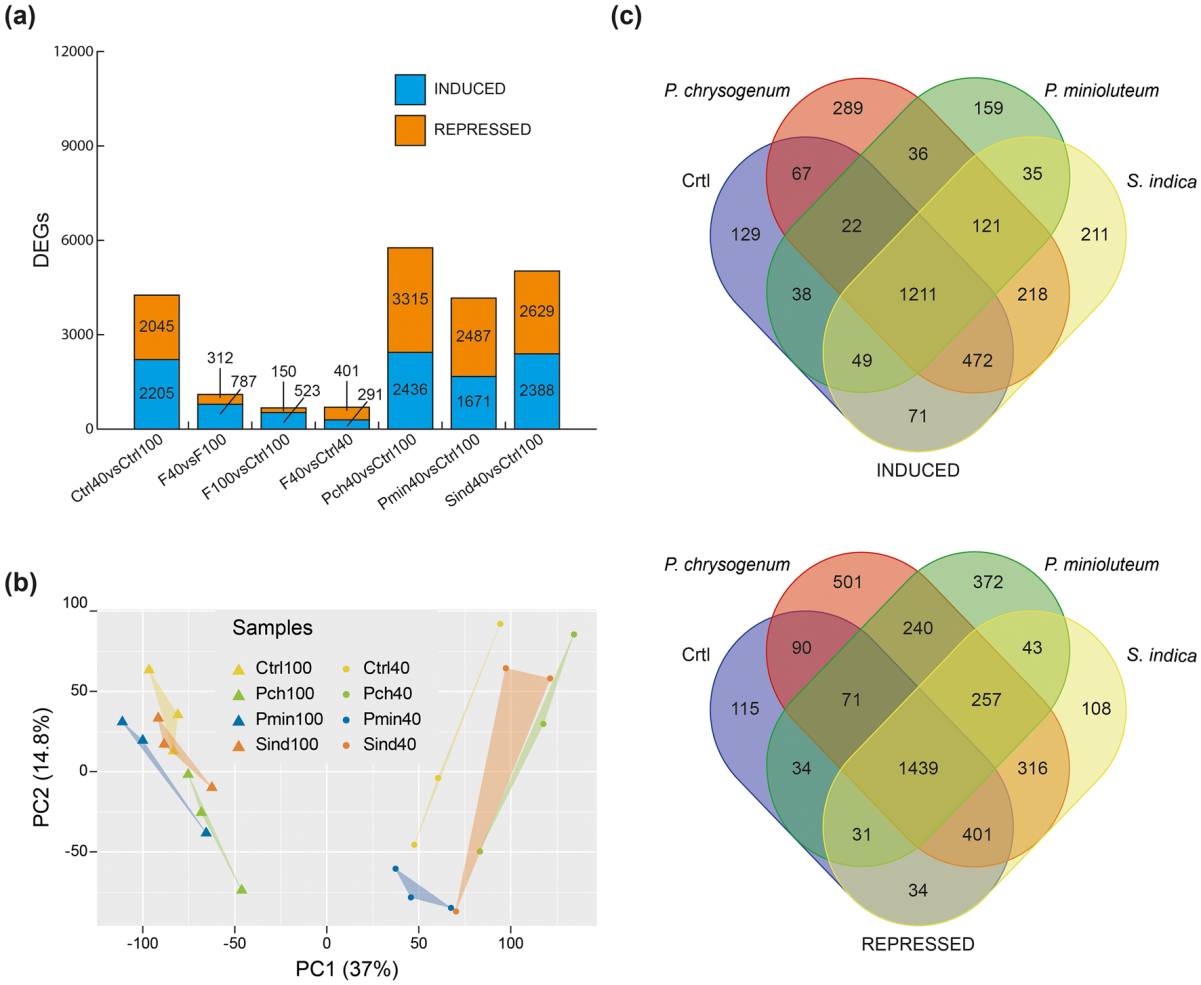
Analysis of the transcriptional alterations caused by fungal inoculations under watered and drought conditions. **a** Bar plots of DEG numbers under drought conditions (40, 40% FC) and watered control conditions (100, 100% FC) in mock treated tomato plants (Ctrl) or fungus inoculated plants (*F* joint fungi, *Pch P. chrysogenum*, *Pmin P. minioluteum*, *Sind S. indica*). **b** Principal component analysis (PCA) of transcriptome-wide normalized gene expression levels assessing the difference between plants inoculated with endophytes and non-inoculated (Ctrl) under drought (circles) and watered (triangles) conditions. The x-axis denotes the PC1; y-axis denotes values for PC2 (**c**) Venn diagrams showing the numbers of DEGs, induced (top panel) and repressed (bottom panel), respectively, of drought stressed and fungus inoculated tomato plants and mock treated control plants (Ctrl) under drought versus the non-inoculated watered condition (Ctrl 100% FC)

Comparison of the transcriptional response of non-inoculated control plants (Ctrl40 vs. Ctrl100) to drought stress with the joint responses of inoculated plants (F40 vs. F100) subjected to identical conditions revealed that the number of DEGs in inoculated plants is substantially reduced compared to non-inoculated plants, suggesting a potential stress-mitigating effect of the fungi. As demonstrated in Fig. 2a, a total of 4250 DEGs were identified for the control plants, whereas only 1099 DEGs were observed in plants co-cultivated with the fungi. Notably, upon applying a threshold of log_2_ (fold change) ≥|1|, the number of DEGs that exhibited a response to the fungal infection under control conditions (F100 vs. Ctrl100) and drought stress conditions (F40 vs. Ctrl40) was relatively low, with 150 and 401 upregulated and 523 and 291 downregulated genes, respectively. The comprehensive analysis of the effects of individual inocula on tomato plants under drought conditions compared to watered reference plants (Ctrl) demonstrated that plants inoculated with *P. chrysogenum* exhibited the highest number of DEGs (5751), followed by plants inoculated with *S. indica* (5017), and *P. minioluteum* (4158). A principal component analysis (PCA) conducted on the normalized read counts (FPKM) of the whole RNA-Seq dataset clearly illustrated the major impact of drought stress. The datasets can be categorized into two groups along the first principal component that explains 37% of the variance, distinctly separating the watered from the drought stress samples (Fig. 2b).

However, no significant difference was observed between inoculated (Pch, Pmin, and Sind) and non-inoculated samples (Ctrl). This observed lack of distinct separation among the groups may be attributed to the experimental design. In an effort to simulate agricultural field conditions, the tomato plants were cultivated in non-sterilized soil within a greenhouse environment with controlled temperature parameters. Nevertheless, this inherent variability, in conjunction with the 6-week interval between root inoculation and tissue collection, may potentially contribute to the observed sample variability. However, at least under drought stress conditions, the *P. chrysogenum* (Pch) and *S. indica* (Sind) samples appear to cluster together and separate from the control plant group.

As illustrated in Fig. 2c, subsequent comparative analysis of the distinct DEG groups under drought conditions revealed the presence of a substantial cohort of DEGs (2650) shared across all inoculated conditions and the non-inoculated condition, comprising 1211 upregulated and 1439 downregulated genes, respectively. This substantial number of shared DEGs indicates a common response to drought conditions in both non-inoculated and inoculated plants. This observation suggests that co-cultivation with the investigated fungi may enhance the molecular mechanism(s) typically activated in response to drought stress. In addition, the analysis highlighted certain DEG groups exclusively shared by fungus-infected plants, which supports the hypothesis of specific responses to fungal infection. Regarding the DEGs shared under all test conditions, it is important to consider that the expression levels of these DEGs may vary significantly between inoculated and non-inoculated conditions. To address this possibility, we conducted an analysis of potential gene expression correlations under drought conditions with the aim of elucidating the mechanisms by which fungal endophytes may enhance drought tolerance in tomato plants.

### Co-expression modules in tomato roots associated with drought and fungus infection

A weighted gene co-expression network analysis (WGCNA) was conducted to examine gene expression patterns under varying conditions, including the presence or absence of endophytes, and under watered or drought conditions.

**Fig. 3:**
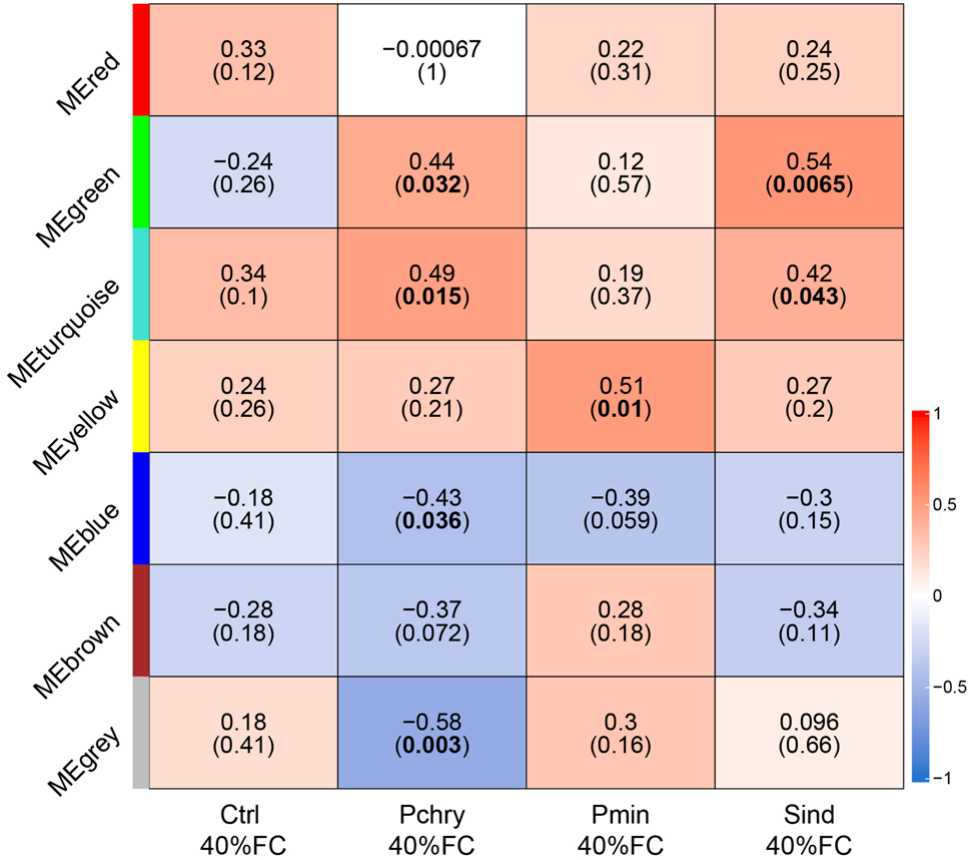
Module trait relationship heatmap for different modules and treatments such as non-inoculated (Ctrl), inoculated with *P. chrysogenum* (Pchry), *P. minioluteum* (Pmin), and *S. indica* (Sind) under drought conditions (40% FC) provided by WGCNA. The upper value in the box indicates the correlation coefficient r between the condition and the module and the value in brackets indicates the *p-value*. This heatmap is colored based on the correlation between the module and the condition: red indicated positive correlations, while blue refers to negative correlations

The WGCNA module trait heatmap, depicted in Fig. 3, demonstrates the identification of seven modules, each denoted by a distinct color. Correlations between modules and conditions with a p-value < 0.05 were deemed statistically significant. The green module exhibited the most significant positive correlation with *P. chrysogenum* (r = 0.44, p = 0.032) and *S. indica* (r = 0.54, p = 0.0065), while the turquoise module displayed a positive correlation with *P. chrysogenum* (r = 0.49, p = 0.015) and *S. indica* (r = 0.42, p = 0.043), and the yellow module with *P. minioluteum* (r = 0.51, p = 0.01). In contrast, negative correlations were observed in the blue module with *P. chrysogenum* (r =  − 0.43, p = 0.036), and the grey module with *P. chrysogenum* (r = 0.58, p = 0.003).

From these correlations, we focused our attention on those that elucidated the primary shared response among the different conditions. We anticipated identifying a module with significant correlations for all tested endophytes. However, as illustrated in Fig. 3, no more than two correlations were observed within a given module. Consequently, we proceeded to examine the correlations in these modules, specifically the green and turquoise modules, and the correlations between inoculations with *P. chrysogenum* and *S. indica*. The turquoise module was ultimately selected due to its higher correlation coefficients and because it also demonstrated a tendency to exhibit a positive correlation with *P. minioluteum*, although this correlation was not statistically significant. Furthermore, it was hypothesized that this module contains gene correlations that could potentially elucidate the more pronounced promotion of shoot biomass production observed in tomato plants inoculated with *P. chrysogenum* compared to those co-cultivated with *S. indica* (Fig. 1b).

**Fig. 4:**
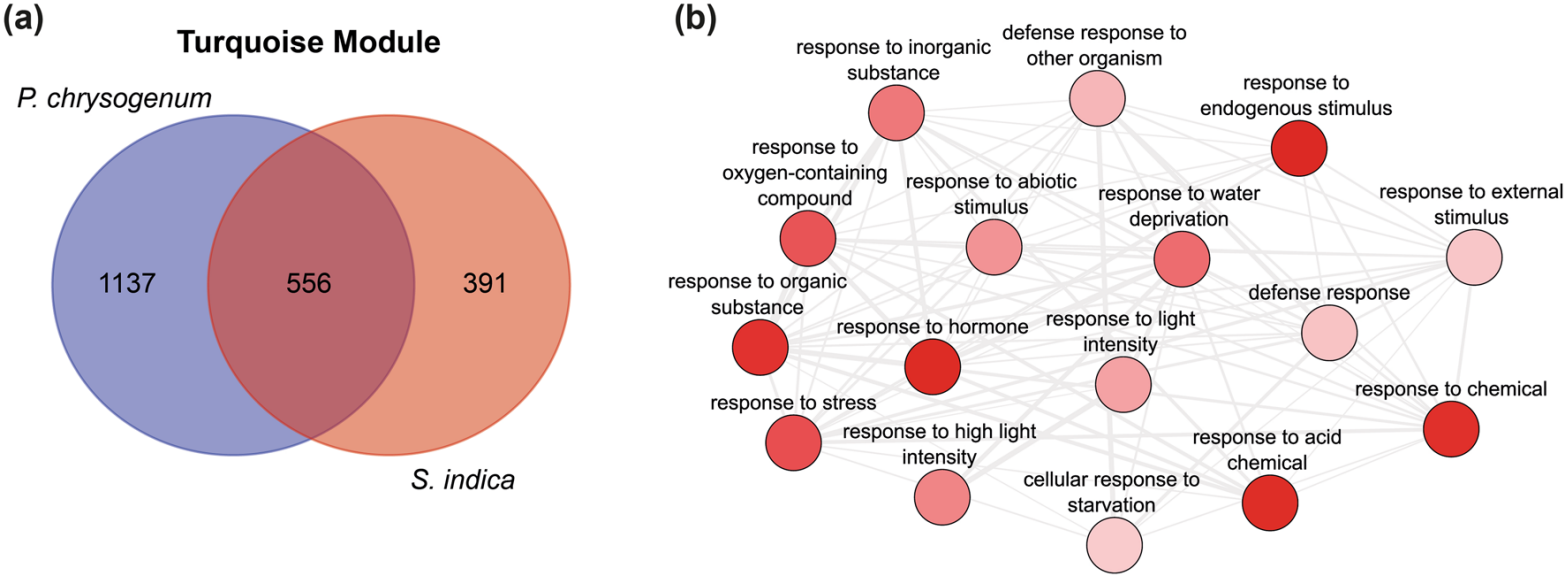
Analysis of the genes in the turquoise module. **a** Venn diagram of tomato genes associated with *P. chrysogenum* and *S. indica* infections extracted from the turquoise module. **b** Functional network analysis of significantly enriched GO:BP terms, after employing ReviGO to reduce redundant GO terms with *A. thaliana* orthologs (darker color refers to lower *p-value*)

Extraction of genes associated with the turquoise module and the inoculated conditions with *P. chrysogenum* and *S. indica* [module eigengene-based connectivity (kME) cut off = 0.5 and gene significance (GS) cut off = 0.4] resulted in the identification of 1693 genes from *P. chrysogenum*-infected plants and 947 genes from *S. indica*-infected plants. Subsequently, these two gene groups were utilized to perform a Venn diagram analysis to identify genes at the intersection of the compared conditions. As depicted in Fig. 4a, 556 genes were found to be shared between the two conditions. To ensure the most comprehensive classification of the genes, we opted to search for the *A. thaliana* orthologs of the 556 tomato genes and conduct the GO enrichment analysis on the Arabidopsis orthologs. A total of 334 Arabidopsis ortholog genes were obtained and employed for the GO analysis.

The GO enrichment analysis revealed multiple overrepresented GO terms (Supplementary Table S2). The most significantly enriched GO:Biological Process (BP) terms were ‘single-organism process’ (GO:0044699), ‘single-organism cellular process’ (GO:0044763), ‘single-organism metabolic process’ (GO:0044710), ‘small molecule metabolic process’ (GO:0044281), ‘metabolic process’ (GO:0008152), ‘response to stimulus’ (GO:0050896), ‘response to endogenous stimulus’ (GO:0009719), ‘organic substance metabolic process’ (GO:0071704) and ‘alpha-amino acid biosynthetic process’ (GO:1,901,607). Following the elimination of redundant GO terms, we focused on a specific cluster of GO terms that includes ‘response to hormone’ (GO:0009725), ‘response to water deprivation’ (GO:0009414), ‘response to acid chemical’ (GO:0001101), ‘response to abiotic stimulus’ (GO:0009628), ‘response to chemical’ (GO:0042221) and ‘defense response’ (GO:0006952), among others (Fig. 4b).

### Core drought stress response module identification in *P. chrysogenum *and *S. indica* inoculated roots

**Fig. 5:**
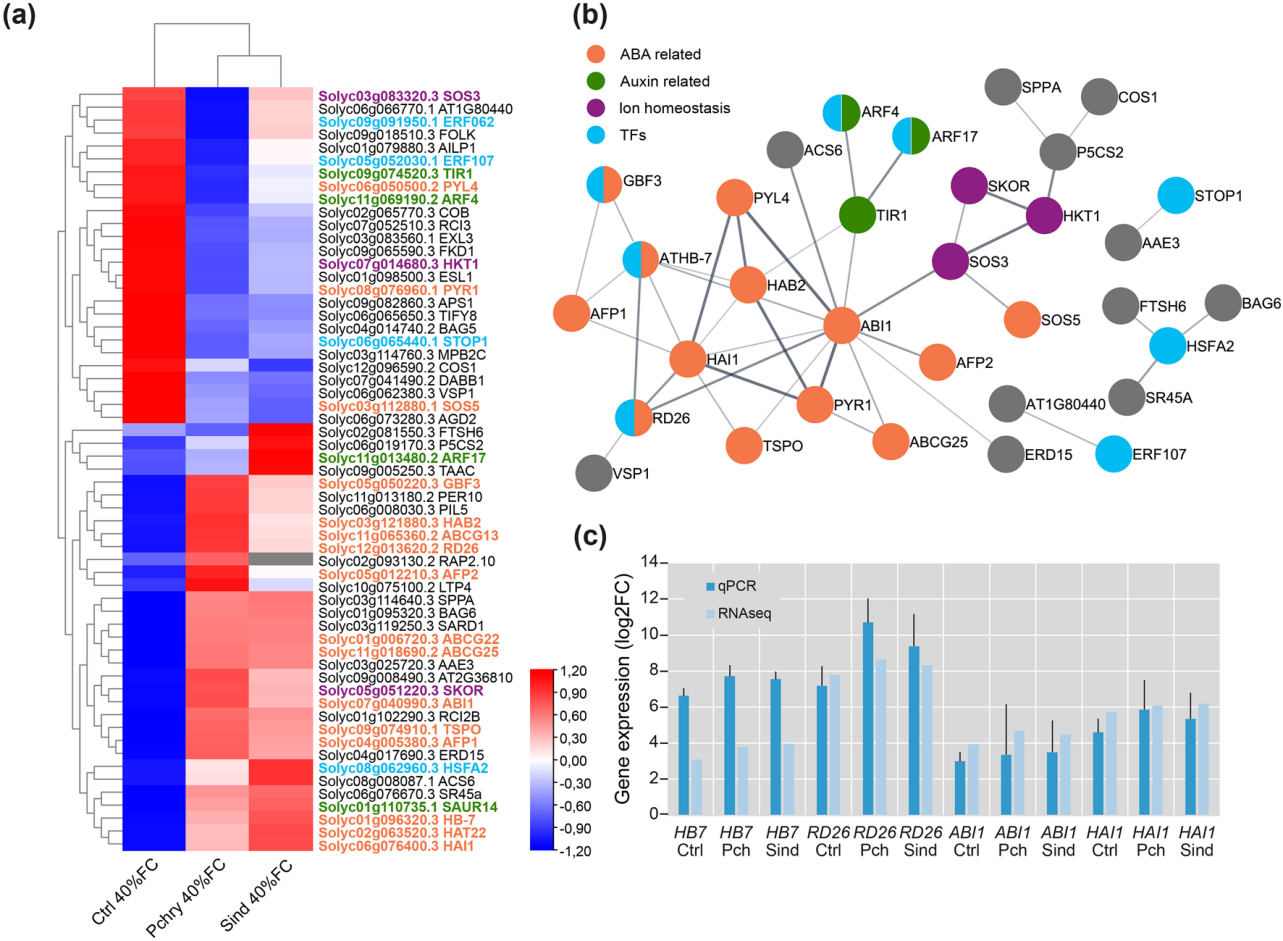
Analysis of genes associated with response to hormone, response to water deprivation and response to abiotic stimulus GO:BP terms. **a** Heatmap showing hierarchical clustering of upregulated and downregulated DEG profiles in non-inoculated tomato plants under drought (Ctrl 40%FC), and tomato plants inoculated with *P. chrysogenum* (Pchry 40% FC) and *S. indica* under drought (Sind 40% FC) compared to non-inoculated tomato plants under well-watered condition (Ctrl 100% FC). **b** Functional interaction network generated using the STRING application and ortholog Arabidopsis genes. Singletons are not presented. **c** Analysis and comparison of *HB7*, *RD26*, *ABI1* and *HAI1* transcriptional alterations by RT-qPCR and RNA-Seq in non-inoculated tomato plants under drought (Ctrl) and tomato plants inoculated with *P. chrysogenum* (Pch) and *S. indica* (Sind), respectively, under drought conditions

With the objective of identifying candidate genes responsible for the enhanced drought tolerance observed in tomato plants inoculated with *P. chrysogenum* and *S. indica*, respectively, 59 genes associated with the GO terms ‘response to hormone’ (GO:0009725), ‘response to water deprivation’ (GO:0009414), and ‘response to abiotic stimulus’ (GO:0009628) were selected to construct a functional interaction network. As illustrated in the heatmap (Fig. 5a), three distinct clusters of genes were identified based on their expression patterns. A group of 26 genes exhibited higher expression in CTRL plants compared to plants inoculated with the fungi. A second group, comprising four genes, demonstrated higher expression in *S. indica* inoculated plants compared to CTRL plants and plants co-cultivated with *P. chrysogenum*. Finally, a third group containing 29 genes was characterized by higher gene expression in the roots of the inoculated plants compared to CTRL plants. The presence of the two large groups supports the hypothesis of a shared drought response in plants inoculated with the two different endophytes.

In the first cluster, we identified genes associated with ABA perception and signaling, including *PYRABACTIN RESISTANCE 1* (*PYR1*), *PYR1-LIKE 4* (*PYL4*), and arabinogalactan protein *SALT OVERLY SENSITIVE 5* (*SOS5*); genes related to auxin perception and signaling such as *TRANSPORT INHIBITOR RESISTANT 1* (*TIR1*) and *AUXIN RESPONSE FACTOR 4* (*ARF4*); transcription factors (TFs) involved in stress response, such as *ETHYLENE RESPONSIVE FACTOR 062* (*ERF062*), *ERF107*, and *SENSITIVE TO PROTON RHIZOTOXICITY 1* (*STOP1*); and two genes associated with jasmonic acid response, namely the phosphatase *VEGETATIVE STORAGE PROTEIN 1* (*VSP1*) and a 6, 7-dimethyl-8-ribityllumazine synthase known as *CORONATINE INSENSITIVE1 SUPPRESSOR 1* (*COS1*). In addition to hormone-related genes, the first cluster comprises two genes associated with ion homeostasis and the salt stress response: *SOS3*, a calcium sensor essential for potassium (K^+^) nutrition and salt expulsion, and *HIGH-AFFINITY K*^+^ *TRANSPORTER 1* (*HKT1*), a sodium (Na^+^) transporter.

In the second cluster, we identified a gene associated with auxin signaling, *ARF17*, and a gene related to proline biosynthesis, *∆*^*1*^*-PYRROLINE-5-CARBOXYLATE SYNTHASE 2* (*P5CS2*).

The third cluster comprises genes involved in ABA signaling, including *ABA INSENSITIVE 1* (*ABI1*), *ABI FIVE BINDING PROTEIN 1* (*AFP1*), *AFP2*, *HOMOLOGY TO ABI2* (*HAB2*), and *HIGHLY ABA-INDUCED 1* (*HAI1*). In addition to genes associated with ABA signaling, this cluster also contains genes related to ABA transport, such as *ATP-BINDING CASETTE G25* (*ABCG25*), *ABCG13* and *ABCG22*, as well as genes involved in ABA response, including *OUTER MEMBRANE TRYPTOPHAN-RICH SENSORY PROTEIN* (*TSPO*) and TFs such as *G-BOX BINDING FACTOR 3* (*GBF3*), *HOMEOBOX 7* (*HB7*), a NAC TF known as *RESPONSIVE TO DESICCATION 26* (*RD26*), and a homeodomain zip (HD zip) class II TF *HAT22*. Furthermore, the third cluster encompassed the potassium transporter *STELAR K*^+^ *OUTWARD RECTIFIER* (*SKOR*) gene, the heat shock factor gene *HEAT SHOCK TRANSCRIPTION FACTOR A2* (*HSFA2*), a gene associated with auxin response *SMALL AUXIN UPREGULATED RNA 14* (*SAUR14*), and a gene involved in ethylene biosynthesis *1-AMINOCYCLOPROPANE-1-CARBOXYLIC ACID SYNTHASE 6* (*ACS6*). Subsequently, we utilized these genes to conduct a functional interaction network analysis to elucidate relationships among the identified genes.

The network analysis confirmed the involvement of several genes in processes related to hormone signaling and biosynthesis. The analysis of the network topology revealed five nodes with considerably high degrees of connectivity: *ABI1* (12 edges), *HAI1* (8 edges), *HB7* (6 edges), *HAB2* (5 edges), and *SOS3* (4 edges) (Fig. 5b). Among these five nodes, three are protein phosphatase 2Cs (PP2Cs) and one is a TF related to ABA responses, emphasizing the significance of ABA in the drought response elicited by the inoculation of *P. chrysogenum* and *S. indica*. The network analysis (Fig. 5b) also highlighted a central core of genes that are potentially responsible for the drought stress response and increased tolerance phenotype exhibited by the plants inoculated with *P. chrysogenum* and *S. indica*. In addition to the ABA-related nodes, the network included three genes associated with ion homeostasis, namely *SOS3*, *HKT1*, and *SKOR*, as well as the three auxin-related genes *TIR1*, *ARF4*, and *ARF17*.

To validate the accuracy of our RNA-Seq data, we conducted a RT-qPCR analysis for 4 of the principal genes identified in the network: *HB7*, *RD26*, *ABI1*, and *HAI1*, and assessed the correlation of the data. As illustrated in Fig. 5c, the data demonstrate substantial correlation for the selected genes under the given test conditions, which supports the robustness of the derived network. The differential expression levels of the genes are presented in Supplementary Table S3.

## Discussion

Beneficial plant symbionts can play crucial roles in mitigating biotic and abiotic stresses in their hosts. In this investigation, we identified a core set of genes that potentially elucidate the observed increase in biomass in aboveground parts of tomato plants under drought conditions when inoculated with the fungal endophytes *P. chrysogenum* and *S. indica*. Numerous studies have previously corroborated the concept of improving drought tolerance in plants, including tomato, through inoculation with root-colonizing fungal endophytes, e.g., *P. chrysoge*num (Morsy et al. 2020; Morales-Quintana et al. 2022), *P. minioluteum* (González-Teuber et al. 2018), and *S. indica* (Sherameti et al. 2008; Tsai et al. 2020; Azizi et al. 2021; Liu et al. 2021; Boorboori and Zhang 2022). These fungi are reported to also increase the tolerance of their host plants to several other abiotic stresses, such as salinity. *Penicillium chrysogenum* in combination with *P. brevicompactum* increases salt stress resistance and plant growth in tomato, lettuce, and cayenne (Molina-Montenegro et al. 2020). *Penicillium minioluteum* enhances salinity resistance in soybeans (Khan et al. 2011), and *S. indica* increases plant resistance to salt stress (Lanza et al. 2019; Boorboori and Zhang 2022). Multiple studies have identified specific physiological, biological, and biochemical mechanisms that contribute to water deficit tolerance in plants induced by fungal endophytes (Dastogeer and Wylie 2017).

This study describes the mutualistic interactions between three distinct fungal endophytes, isolated from unrelated arid locations, and the agriculturally significant crop plant, *S. lycopersicum*. All three fungal symbionts conferred increased drought tolerance to tomato plants, as evidenced by the significantly higher biomass production of the aerial plant parts of seedlings infected with the fungi compared to the non-infected control plants (Fig. 1b). To elucidate whether the tested root colonizing endophyte fungi triggered the same molecular mechanism(s) to confer increased drought tolerance, a comprehensive RNA-Seq analysis was conducted on tomato roots inoculated and non-inoculated with the endophytes under two different water regime conditions. As illustrated in Fig. 2a, the number of DEGs was greater in the comparison between non-inoculated plants (Ctrl 40% FC vs. Ctrl 100% FC) subjected to drought stress and fungus-inoculated plants (Fungi 40% FC vs. Fungi 100% FC) under similar conditions. This observation indicates a possibly attenuated stress response in tomato plants co-cultivated with the tested fungi. Furthermore, the analysis of the transcriptional effect of the fungal infections under control (Fungi 100% FC vs. Ctrl 100% FC) and drought conditions (Fungi 40% FC vs. Ctrl 40% FC) revealed only a minimal impact on gene expression levels. It is noteworthy that the number of DEGs for *P. chrysogenum* and *S. indica* under combined stress conditions (Pch 40% FC vs. Ctrl 100% FC, 5751 genes; Sind 40% FC vs. Ctrl 100% FC, 5017 genes) significantly exceeds the sum of the individual stress responses (Ctrl 40% FC vs. Ctrl 100% FC, 4259 genes; Fungi 100% FC vs. Ctrl 100% FC, 673 genes). This observation is likely attributable to an extensive reprogramming of the drought stress response when the plants were infected with the different symbionts. The response to *P. minioluteum*, however, was less pronounced, which separates this fungus from the two others. As demonstrated by the PCA (Fig. 2b), the highest degree of explained variability in the RNA-Seq data is associated with drought conditions, as reflected in the first principal component, which substantially corroborates the significant impact of drought on the transcriptome. Our hypothesis that the fungi could induce general drought stress response modules in tomato was supported by the absence of clear differentiation between control samples and those of fungus-inoculated plants. To gain deeper insight into shared transcriptomic alteration between drought stress and combined drought stress and individual fungus infections, we conducted a Venn diagram analysis to elucidate intersections among the set of DEGs from control and inoculated roots under drought conditions compared to non-inoculated roots under well-watered conditions (Fig. 2c). In this plot, the highest number of DEGs is shared between the inoculated and the non-inoculated conditions, indicating the existence of a large gene cluster related to drought stress response in tomato roots, regardless of the inoculation condition. However, this analysis did not adequately account for the possibility that the interaction with the symbionts merely potentiates the response of genes that are also induced under control conditions, albeit at a lower level. In this context, to further investigate the transcriptional analysis, a WGCN analysis was performed. The WGCNA examined the correlation patterns among DEGs under various inoculation and drought conditions (Fig. 3). In the absence of a module showing a significant correlation among all three inoculated conditions, we rejected the hypothesis of a shared common response to drought among *P. chrysogenum*, *P. minioluteum*, and *S. indica*-inoculated roots. However, we identified the turquoise module, which exhibited a significant correlation under conditions in which the plants were inoculated with either *P. chrysogenum* or *S. indica*. This observation highlights a common shared drought response between tomato plants inoculated with the two fungi. With regard to the RNA-Seq data obtained for *P. minioluteum*, it can be inferred that the fungus-induced drought tolerance enhancement of the tomato plants is likely achieved through an alternative mechanism, or by a more subtle modification of the transcriptome. The latter possibility is suggested by the positive, albeit statistically non-significant, correlation with the fungus. The correlation between this fungus and the two others was not significant, although the tomato plants inoculated with *P. minioluteum* demonstrated a similar increased biomass production under drought stress compared to those infected with the other two symbionts. It will be an intriguing future endeavor to elucidate the complex network of transcriptional alterations triggered by *P. minioluteum* in tomato plants under drought stress conditions and compare them with those responses observed for the two other fungi. However, given the substantial correlation between the responses triggered by *P. chrysogenum* and *S. indica*, we focused our investigation on the comparison of the interactions between those two fungi and tomato.

**Fig. 6:**
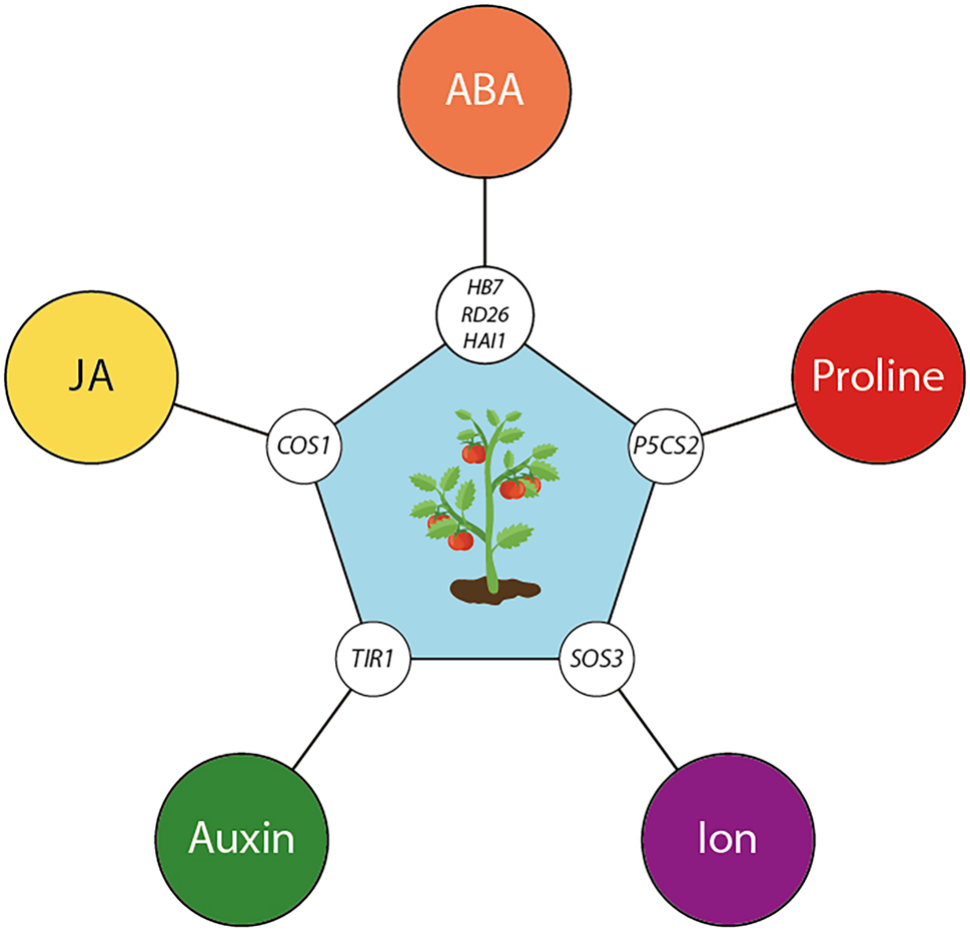
A model summarizing the core drought response modules identified in this work

With the objective of gaining a more comprehensive understanding of the shared processes elicited in drought-stressed tomato plants co-cultivated with *P. chrysogenum* and *S. indica*, we conducted further analysis of the associated genes found in the turquoise module (Fig. 4a). To enhance the scope of the analysis and maximize the information extracted from the dataset, we utilized *A. thaliana* ortholog genes, leveraging Arabidopsis as the most well-developed model for translational research in plants (Yaschenko et al. 2024). The GO analysis (Fig. 4b) revealed GO terms of particular interest, namely ‘response to hormone’ (GO:0009725), ‘response to water deprivation’ (GO:0009414), and ‘response to abiotic stimulus’ (GO:0009628), as they suggested a direct connection to genes associated with biological functions related to responses to drought stress and that were differentially expressed in the presence of fungi. The selected GO classifications encompassed 59 associated genes. To further investigate the differential regulation of these genes, we analyzed their expression in non-infected control plants (Ctrl) and in plants infected with either *P. chrysogenum* or *S. indica* under drought stress conditions (Fig. 5a). Through the application of a hierarchical clustering approach, we identified three clusters based on the growth conditions and the expression levels of the genes. Based on the observed results, we conclude that the shared response to drought stress in plants infected with fungi is mediated by the same group of genes. However, it is noteworthy that the expression levels differ between *P. chrysogenum and S. indica* inoculated plants. These differences in expression levels may potentially account for the variations in the observed phenotypes. Plants inoculated with *P. chrysogenum* exhibited higher biomass compared to those inoculated with *S. indica* when grown under drought stress conditions (Fig. 1b). The identified genes were subsequently utilized to construct a functional interaction network with the aim of identifying a core regulatory module within the 59 candidate genes (Fig. 5b). The resulting network provided evidence for the existence of several gene clusters that share common biological functions, which appear to be regulated by a reduced subset of genes. Our findings are summarized in the model presented in Fig. 6, which illustrates the proposed main drivers of the increased drought tolerance phenotype observed in the tomato plants co-cultivated with either *P. chrysogenum* or *S. indica*.

The largest group of nodes is associated with ABA-dependent processes. This observation aligns with several reports highlighting the pivotal role of ABA in enhancing resistance to drought stress in plants harboring *S. indica* in their roots (Peskan-Berghöfer et al. 2015; Xu et al. 2017). Furthermore, 4 out of the 5 nodes with the highest degree of connectivity in the network are ABA-related, underscoring its crucial role in conferring increased drought stress tolerance in plants interacting with *P. chrysogenum* or *S. indica*. Among the 13 ABA-related genes, we identified several TFs, including *GBF3*, *HB7*, and *RD26*, as well as 5 PP2Cs, namely *ABI1*, *AFP1*, *AFP2*, *HAB2*, and *HAI1*. An induction of *GBF3* is reported to confer increased resistance to drought in *A. thaliana* (Ramegowda et al. 2017). The ectopic expression of *HB7* has also been demonstrated to increase drought tolerance in tomato (Mishra et al. 2012), while the induced expression of *RD26* is similarly known to enhance drought tolerance in plants (Nakashima et al. 2012; Duan et al. 2019). These three TFs appear to be transcriptionally activated in the roots of tomato plants that established a symbiosis with *P. chrysogenum* or *S. indica* under drought conditions compared to non-inoculated roots from control plants grown under drought conditions (Supplementary Table S3). Consequently, we conclude that these TFs are critical molecular components for the fungus-triggered drought tolerance mechanism in tomato plants.

The promotion of proline biosynthesis is a well-established drought stress response. Proline serves critical functions as an osmoregulator, chemical chaperone, and ROS scavenger (Liang et al. 2013). Our network analysis revealed the induction of *P5CS2*, a key gene in proline biosynthesis. ∆^1^-pyrroline-5-carboxylate synthases (P5CSs) catalyze the conversion of glutamate to γ-glutamate-semialdehyde, and they are closely associated with osmotic and drought stress responses (Amini et al. 2015). The induction of *P5CS1* and *P5CS2* in Arabidopsis is mediated by ABA (Strizhov et al. 1997), suggesting a potential correlation between the ABA gene group and *P5CS2* (Fig. 5b). Furthermore, it has been demonstrated that *S. indica* infections stimulate the accumulation of several metabolites in tomato, including betaine, glycine, and proline (Ghorbani et al. 2018), and induce *P5CS* gene expression under drought conditions (Azizi et al. 2021).

In tomato, *SOS* genes are involved in salt tolerance mechanisms (Huang et al. 2024). Specifically, in response to increases in cytoplasmic Ca^2+^ concentration, the Ca^2+^ sensor SOS3 is activated, which facilitates interaction with the kinase SOS2. Subsequently, the SOS3/SOS2 complex phosphorylates and thereby activates the plasma membrane Na^+^/H^+^ antiporter SOS1, resulting in reduced Na^+^ toxicity in plant cells (Martínez-Atienza et al. 2007). Recently, SOS3 has also been demonstrated to interact with the Na^+^ transporter HKT1 (Gámez-Arjona et al. 2024). Our RNA-Seq data provided evidence for a more pronounced repression of *SOS3* and *HKT1* in *P. chrysogenum* and *S. indica* inoculated roots under drought conditions compared to the non-inoculated roots under drought conditions (Supplementary Table S3). This observation suggests that the inoculated plants may accumulate more Na^+^ through the reduced abundance of SOS1 and HKT1. The regulation of Na^+^ uptake and compartmentation has been reported to contribute to the preservation of cell turgor (Álvarez-Aragón and Rodríguez-Navarro 2017). As illustrated in Fig. 5a, the gene encoding the potassium channel *SKOR* exhibits stronger induction in the *P. chrysogenum* and *S. indica* inoculated roots than in the non-inoculated roots. This potentially increases the delivery of K^+^ from the stellar cells to the xylem (Liu et al. 2006), leading to higher K^+^ accumulation in aboveground parts of the plant. This observation aligns with the previously described impact of *S. indica* on the distribution of K^+^ in Arabidopsis (Pérez-Alonso et al. 2022). Consequently, this phenomenon may influence the regulation of stomatal aperture.

In the plant hormone-related groups, *TIR1* appears to be more strongly repressed in the inoculated roots than in the non-inoculated roots. TIR1 is part of the SCF^TIR1/AFBs^-Aux/IAA [SKP-Cullin-F box (SCF), TIR1/AFB (AUXIN SIGNALING F-BOX), AUXIN/INDOLE ACETIC ACID (Aux/IAA)] complex, thus forming part of the canonical auxin perception machinery (Salehin et al. 2015). The loss of *TIR1* in Arabidopsis is reported to increase drought tolerance (Salehin et al. 2019). Along with the repression of *TIR1*, we were also able to detect the stronger repression of the jasmonate (JA)-related gene *COS1*. JA has a proven role in biotic and abiotic stress responses (Wang et al. 2021). *COS1* acts as a *CORONATINE INSENSITIVE 1* (*COI1*) suppressor that is essential for JA perception and the regulation of JA-mediated plant defense and senescence (Xiao et al. 2004). It will be an important future task to investigate these findings through reverse genetics experiments.

In conclusion, we propose a molecular mechanism, shared by *P. chrysogenum* and *S. indica*, that involves the transcriptional regulation of a central core module of genes, responsible for the increased drought tolerance phenotype of tomato plants inoculated with the fungal endophytes. In this context, it is noteworthy that the two endophytes were isolated from two geographically distinct desert environments but appear to trigger the same conserved gene cluster in a non-specific host plant that is not endemic to the habitats where the fungi were isolated. This observation strongly supports the hypothesis that the tested root-colonizing beneficial fungi acquired the required properties to increase drought tolerance in plants by addressing highly conserved mechanisms in plants independently from each other over the course of coevolution with their host plants in similar extreme environments.

## Supplementary Information

Below is the link to the electronic supplementary material.

Supplementary file1 (JPG 9929 KB) ** Supplementary Fig. S1** Tomato root infection and re-isolation experiment of the examined root-colonizing plant endophytic fungi

Supplementary file2 (JPG 1840 KB) **Supplementary Fig. S2** Analysis of stomatal conductance and shoot fresh weight of tomato plants 6 weeks after mock (Ctrl) and fungus treatments

Supplementary file3 (XLSX 10 KB)

Supplementary file4 (XLSX 13 KB)

Supplementary file5 (XLSX 12 KB)

## Data Availability

All data supporting the findings of this study are available within the paper and within its Supplementary Data published online. The RNA-Seq data produced in this work are openly available in Gene Expression Omnibus under the code GSE279454.
