# Supplementary figures and images for: Identification of a drought stress response module in tomato plants commonly induced by fungal endophytes that confer increased drought tolerance
Source: Plant Mol Biol. 2024 Dec 17;115(1):7. doi: 10.1007/s11103-024-01532-y (PMC11652604; doi:10.1007/s11103-024-01532-y)

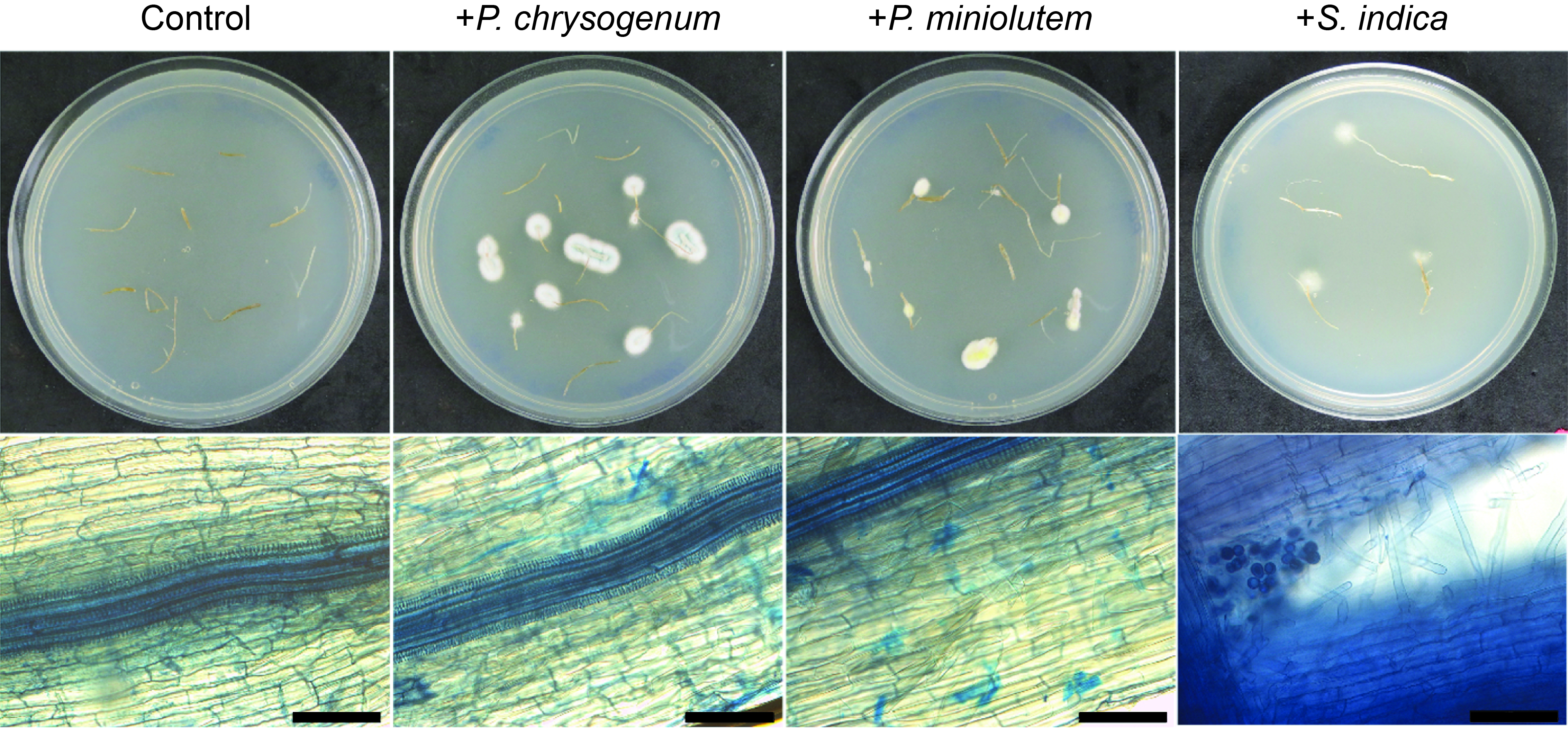

Supplement: Supplementary file 1 — Supplementary file1 (JPG 9929 KB) Supplementary Fig. S1 Tomato root infection and re-isolation experiment of the examined root-colonizing plant endophytic fungi [file 11103_2024_1532_MOESM1_ESM.jpg]

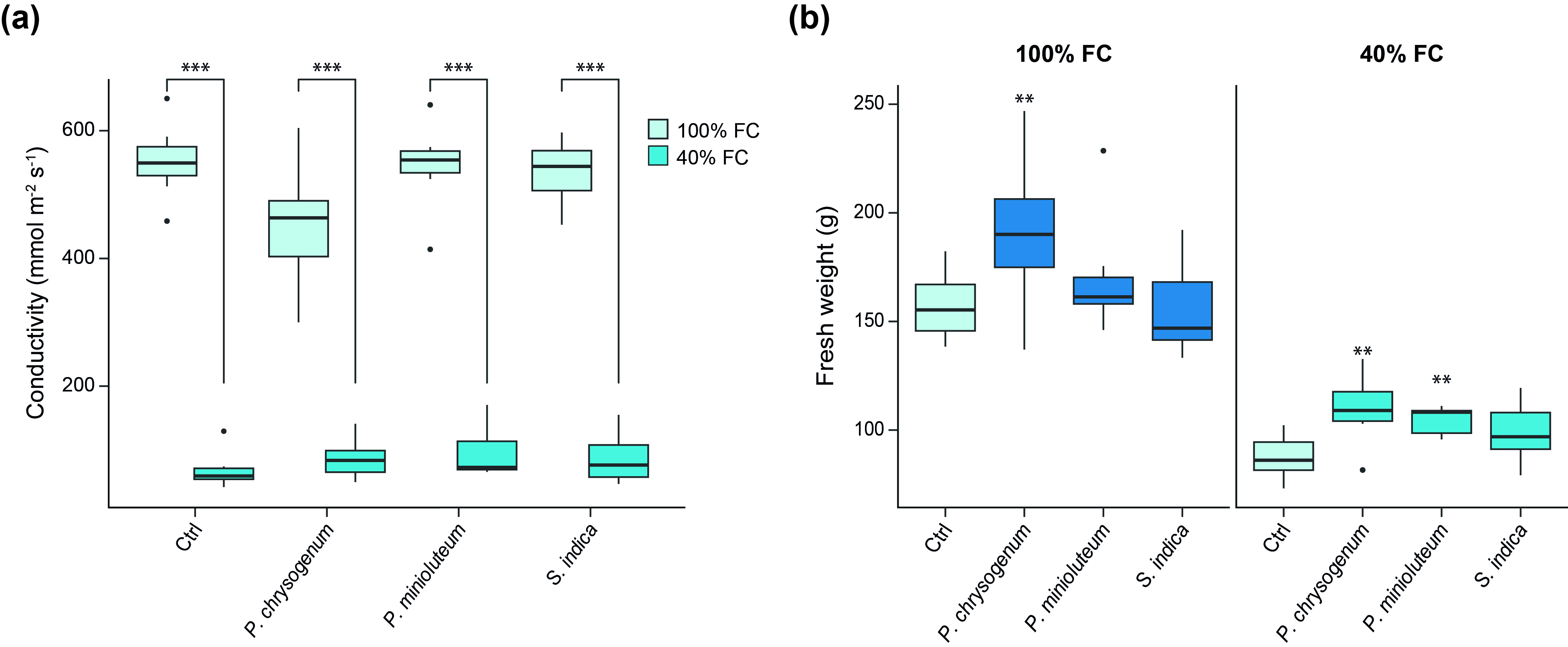

Supplement: Supplementary file 2 — Supplementary file2 (JPG 1840 KB) Supplementary Fig. S2 Analysis of stomatal conductance and shoot fresh weight of tomato plants 6 weeks after mock (Ctrl) and fungus treatments [file 11103_2024_1532_MOESM2_ESM.jpg]
